# Genomes and virulence difference between two physiological races of *Phytophthora nicotianae*

**DOI:** 10.1186/s13742-016-0108-7

**Published:** 2016-01-28

**Authors:** Hui Liu, Xiao Ma, Haiqin Yu, Dunhuang Fang, Yongping Li, Xiao Wang, Wen Wang, Yang Dong, Bingguang Xiao

**Affiliations:** CAS-Max Planck Junior Research Group, State Key Laboratory of Genetic Resources and Evolution, Kunming Institute of Zoology, Chinese Academy of Sciences, Kunming, 650223 China; University of Chinese Academy of Sciences, Beijing, 100049 China; Yunnan Agricultural University, Kunming, 650100 China; Yunnan Academy of Tobacco Agricultural Sciences, Yuantong Street No.33, Kunming, Yunnan 650021 China; Faculty of Life Science and Technology, Kunming University of Science and Technology, Kunming, 650500 China

**Keywords:** Black shank, *Phytophthora nicotianae*, Genomes, Hybrid assembly, RxLR effector

## Abstract

**Background:**

Black shank is a severe plant disease caused by the soil-borne pathogen *Phytophthora nicotianae*. Two physiological races of *P. nicotianae*, races 0 and 1, are predominantly observed in cultivated tobacco fields around the world. Race 0 has been reported to be more aggressive, having a shorter incubation period, and causing worse root rot symptoms, while race 1 causes more severe necrosis. The molecular mechanisms underlying the difference in virulence between race 0 and 1 remain elusive.

**Findings:**

We assembled and annotated the genomes of *P. nicotianae* races 0 and 1, which were obtained by a combination of PacBio single-molecular real-time sequencing and second-generation sequencing (both HiSeq and MiSeq platforms). Gene family analysis revealed a highly expanded ATP-binding cassette transporter gene family in *P. nicotianae*. Specifically, more RxLR effector genes were found in the genome of race 0 than in that of race 1. In addition, RxLR effector genes were found to be mainly distributed in gene-sparse, repeat-rich regions of the *P. nicotianae* genome.

**Conclusions:**

These results provide not only high quality reference genomes of *P. nicotianae*, but also insights into the infection mechanisms of *P. nicotianae* and its co-evolution with the host plant. They also reveal insights into the difference in virulence between the two physiological races.

**Electronic supplementary material:**

The online version of this article (doi:10.1186/s13742-016-0108-7) contains supplementary material, which is available to authorized users.

## Data description

### Background

*Phytophthora nicotianae*, also known as *Phytophthora parasitica* var. *nicotianae*, is a soil-borne bi-flagellated oomycete plant pathogen, which causes black shank in cultivated tobacco (*Nicotiana tabacum*), and root rot, leaf necrosis, and stem lesions in a variety of plants [1]. *P. nicotianae* is able to infect a wide range of hosts, spanning 255 genera in 90 different plant families. It devastates the production of a number of economically important plants, and causes millions of dollars worth of economic losses each year in the tobacco industry alone [2, 3]. So far, management strategies for *P. nicotianae* are limited to non-host crop rotation, cultivation of pathogen-resistant breeds, and the use of chemical control (e.g. mefenoxam) [4]. The primary reason for the difficulty in controlling *P. nicotianae* is the production and survival of chlamydospores in unfavorable growth conditions, as well as the production of motile zoospores. The ability of *P. nicotianae* to infect specific tobacco cultivars with different resistance genes defines four physiological races (0, 1, 2 and 3). The predominant physiological races, 0 and 1, are widely distributed throughout China, the United States and other major tobacco cultivation areas [5, 6]. Previous studies using tobacco cultivars with moderate or high levels of resistance have found that race 0 has better pathogenic and ecologic fitness levels than race 1, suggesting that the difference in virulence between the two races is affected by additional genetic factors [7]. To discover better and more efficient ways to control the pathogen, we undertook a global examination of the genes involved in the infection process from different races. Although five strains of *P. parasitica* are already public available [8], none of these includes any of the four physiological races of *P. nicotianae*. Here we report the genomes of *P. nicotianae* physiological races 0 and 1, sequenced using a combination of PacBio single-molecule real-time (SMRT) sequencing technology, and Illumina HiSeq and MiSeq sequencing technologies, and identify candidate genes that may cause the difference in virulence between them.

### Isolation *of P. nicotianae* races and genomic DNA extraction

Tobacco plants infected by either *P. nicotianae* race 0 or race 1 were obtained from Yunnan Tobacco Research Institute. Any surface dirt on the infected plant was washed off under tap water. After drying, stem tissue from the lesion margin were cut into 5 × 5 mm squares, sterilized using 70 % ethanol for 1 minute, and then rinsed three times using sterile water. Sterilized tissue squares were then placed in lima bean agar (LBA) plates amended with 50 μg/ml ampicillin, 100 μg/ml rifampicin, and 50 μg/ml of pentachloronitrobenzene to suppress possible contaminant. LBA plates were incubated for 2–3 days in darkness at 25 °C. Color and texture of the colony and mycelium were used to confirm the identity of *P. nicotianae*. Mycelium was transferred to LBA slants and cultured for 7 days in darkness at 25 °C. Genomic DNA was extracted using the modified cetyltrimethyl ammonium bromide method [9].

### Sequencing and quality control

Whole-genome sequencing yielded 41 Gb HiSeq paired-end reads, 5 Gb HiSeq mate pair reads, 5 Gb MiSeq reads, and 5 Gb PacBio long reads for race 0; and 46 Gb HiSeq pair-end reads, 4 Gb HiSeq mate pair reads, 3 Gb MiSeq reads, and 6 Gb PacBio long reads for race 1 (Table 1). Illumina HiSeq reads were first filtered out with >10 % N or with >40 bp low quality bases. Redundant reads resulting in duplicate base calls were filtered at a threshold of a Euclid distance ≤ 3 and a mismatch rate of ≤ 0.1. Where duplicated paired-end (PE) reads were identical, only one copy was retained. For adapter contamination caused by DNA adapter dimerization, empty loading, or too small an insert size (less than a read length), we filtered out if both read 1 and read 2 contained an adapter ≥ 10 bp with a mismatch rate ≤ 0.1. For PacBio reads, we first used the HGAP (SMRT Analysis v2.1.1) pipeline to perform self-correction (default parameters). Longer PacBio reads were selected automatically as seeds; the rest of the reads were aligned against these seed sequences for correction. For hybrid correction, we used LSC [10] (v1.0 alpha) with the parameter for bowtie2 set to *very-fast*; pacBioToCA [11] (wgs v8.0) using the parameter *length 500*. Corrected PacBio long reads were obtained by aligning high accuracy HiSeq short reads against PacBio long reads. We also used ECTools (July 6^th^ 2014) to correct PacBio long reads from both *P. nicotianae* races 0 and 1. ECTools aligned unitigs assembled from MiSeq reads against PacBio long reads to perform correction.

**Table 1:** Sequencing and data size of *P. nicotianae* races 0 and race 1

Races	Library type	Instrument	Fragment size (bp)	Read length (bp)	Data (Gb)	
					Before quality control	After quality control
*P. nicotianae* race 0	Illumina paired-end	Hiseq	350	100	41	34
	Illumina mate pair	Hiseq	2,000	100	5	3
	Illumina paired-end	Miseq	500	~300	5	^a^3
	SMRTbell	PacBio RS	10,000	~1,932	5	^b^3
*P. nicotianae* race 1	Illumina paired-end	Hiseq	350	100	46	20
	Illumina mate pair	Hiseq	2,000	100	4	2
	Illumina paired-end	Miseq	500	~300	3	^a^2
	SMRTbell	PacBio RS	10,000	~2,333	6	^b^4

^a^Total base pairs of Miseq reads after merged using Flash

^b^PacBio reads after correction with LSC

### Assembly

**Fig. 1:**
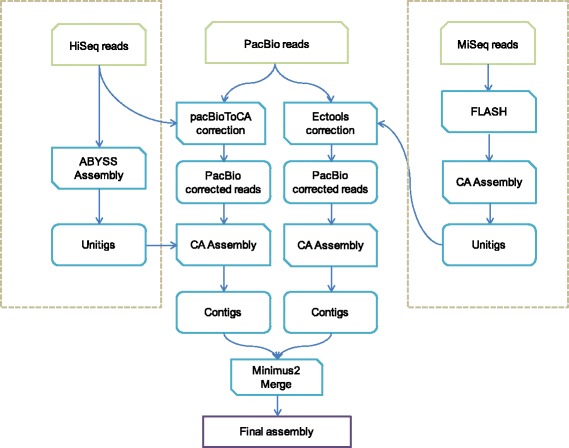
Assembly pipeline for *P. nicotianae* race 0. CA indicates Celera Assembler. Because of high heterozygosity of spores in *P. nicotianae* race 0, we used a hybrid approach including Celera Assembler, ABySS assembler, and Minimus2 to assemble this genome

**Fig. 2:**
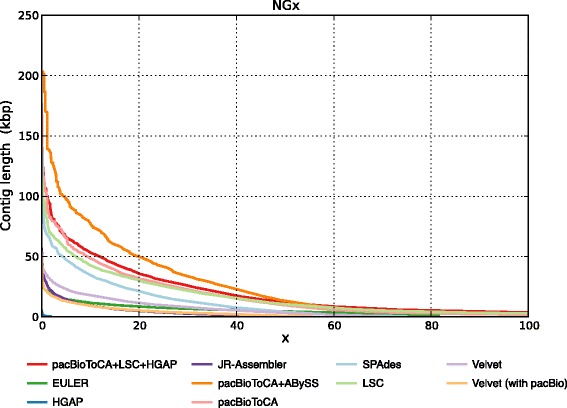
NGx plot for different assemblies. Contigs of length equal to or above NGx occupy x % of the reference genome. pacBioToCA + LSC + HGAP refers to assembly from non-redundant corrected PacBio reads of LSC, pacBioToCA and HGAP. pacBioToCA + ABySS means assembly using pacBioToCA corrected reads and unitigs assembled by ABySS. Assembly from pacBioToCA + ABySS was also merged with assembly from ECTools corrected PacBio reads to generate the final assembly, which was then used for annotation. As illustrated, the NG50 for hybrid assemblies such as pacBioToCA + ABySS were longer

Because of its relatively high heterozygosity, we used a hybrid assembly approach to assemble the genome of race 0 (Fig. 1). We also compared the performance of different assemblers including Velvet (v1.2.09) [12], ABySS (v3.81) [13], JR-Assembler (v1.0.3) [14], EULER-SR (v1.1.2) [15], SPAdes (v3.0.0) [16], SOAPdenovo2 (r240) [17], Celera Assembler (v8.0) [18] and Minimus2 (v 3.1) [19] on *P. nicotianae* race 0. Comparison showed that assemblies from PacBio reads were generally of better quality than those from HiSeq reads (Fig. 2). The final assembled genome sizes for race 0 and race 1 were 80 Mb and 69 Mb, respectively, which is slightly different from the previous estimation of *P. nicotianae* (90 Mb) [20]. The corresponding contig N50 sizes were 23 kB and 30 kB, respectively (Table 2). Over 95 % of core eukaryotic genes could be mapped to the two genomes using CEGMA [21], and over 90 % of Illumina HiSeq reads could be mapped back to the genome assemblies.

**Table 2:** Genome and gene statistics of *P. nicotianae* races 0 and race 1

Races	Categories	Number	N50 (kb)	Longest size (kb)	Size (Mb)	Percentage of the assembly
*P. nicotianae* race 0	Total contigs	6142	23	203	81	-
	Total scaffolds	6139	23	203	81	-
	Genes annotated	17,797	1.8	26	27	33 %
	Transposable elements	-	-	-	32	39 %
*P. nicotianae* race 1	Total contigs	6116	22	196	71	-
	Total scaffolds	5317	30	318	71	-
	Genes annotated	14,542	1.7	15	20	28 %
	Transposable elements	-	-	-	27	38 %

### Annotation

Known transposable elements (TEs) were identified with RepeatMasker (version 3.2.6) [22] using the Repbase TE library (v16.10) [23] and default parameters. Tandem repeats were predicted using TRF [24]. *gypsy* and *copia* types of long terminal repeat (LTR) were the main contributors to the repeat, making up 12.5 % and 3.5 % of the genome for race 0, and 11.5 % and 3.6 of the genome for race 1. For gene structure prediction, gene sets from 9 species including *Phytophthora infestans* [25], *Phytophthora sojae* [26], *Phytophthora ramorum* [26], *Hyaloperonospora arabidopsis* [27], *Pythium aphanidermatum* [28], *Pythium arrhenomanes* [28], *Pythium irregulare* [28], *Pythium vexans* [28], *Pythium iwayamai* [28] and *Pythium ultimum* [29] were used for homology-based prediction. GENSCAN [30], AUGUSTUS [31] and GlimmerHMM [32] were used for *de novo* gene prediction. Evidence derived from homology-based and *de novo* predictions were then integrated in GLEAN to generate a consensus gene set. A total of 17,797 and 14,542 protein-coding genes were annotated in *P. nicotianae* race 0 and race 1, respectively. Over 97 % of these genes could be aligned against KEGG [33], Swiss-Prot and TrEMBL databases [34]. Mean exon numbers per gene in *P. nicotianae* and related species varied between 2.2 and 2.8, suggesting that homology and *de novo*-based prediction were appropriate for annotation (Additional file 1). We also used publicly available expressed sequence tags (ESTs) from the appressorium [35] and mycelium [36, 37] of *P. nicotianae* to validate the annotation. We retrieved a total of 10,524 ESTs from the dbEST database. Using the threshold of match length >200 bp and E-value <1e-5, we aligned 8,043 ESTs to the race 0 genome and 7,618 ESTs to the race 1 genome. Additionally, 4,454 genes in race 0 and 3,604 genes in race 1 were supported by at least one EST (Additional file 2). Whole genome comparison using NUCmer [38] found that average identity was 99 % for 1-to-1 alignment, and 98.84 % for m-to-m alignment between *P. nicotianae* races 0 and 1. Using KaKs_Calculator, mean synonymous mutation ratio (*Ks*) was estimated to be 0.075 between race 0 and race 1 [39], and four genes were identified to be positively selected (Additional file 3).

### Gene family clustering and evolution

**Fig. 3:**
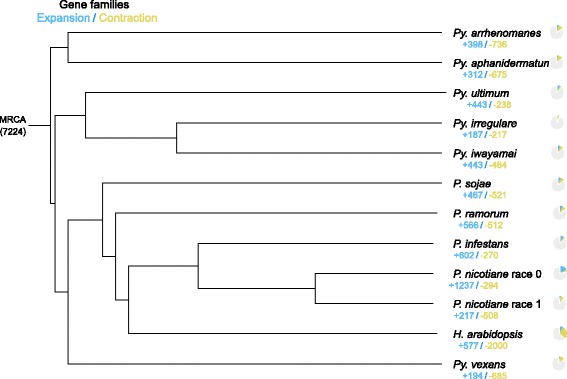
Gene family expansion and contraction in *P. nicotianae* races 0 and 1, and related species. The proportions of expanded (blue) and contracted (yellow) gene families are shown as pie charts at each branch terminus. MRCA represents most recent common ancestor

Gene family clustering using OrthoMCL [40] revealed that over 72 % of gene families were shared between species pairs among *P. nicotianae* race 0, race 1, and related species (Additional file 4). The average number of genes per gene family was 1.19 to 1.50 in *Phytophthora* and 1.14 to 1.26 in *Pythium*, suggesting more copies of genes exist in the *Phytophthora* genus. A total of 1,604 single-copy genes were identified between *P. nicotianae* and the other 9 related species (Additional file 5). Gene family expansion and contraction estimated using CAFÉ [41] found that 1,237 gene families expanded and 294 gene families contracted in race 0, while 217 gene families expanded and 508 gene families contracted in race 1 (Fig. 3).

### ABC transporter expanded in *P. nicotianae*

The ATP-binding cassette transporter (ABC transporter) superfamily facilitates the transport of ions, proteins, lipids and toxins across plant membranes [42]. Interestingly, a domain-centric study found this gene family to be enriched in the oomycete plant pathogen genomes [43]. It was proposed that an important function of ABC transporters in pathogens involves exporting toxic phytoalexins [44, 45]. Based on the result of CAFÉ analysis, we found the ABC transporter gene family to be significantly expanded in the branch of *P. nicotianae* (likelihood ratio test, *p*-value < 0.05), but not in the branch of *P. infestans* (likelihood ratio test, *p* = 0.9). To verify this result, we used Pfam to annotate ABC transporter domains (PF00005.22, PF00664.18, PF01061.19) between *P. infestans*, and *P. nicotianae* races 0 and 1 (Additional file 6). The portions of ABC transporters in *P. nicotianae* were significantly larger than that in *P. infestans* (chi-square test, *p* < 0.05). This result suggests that the ABC transporter family plays important roles in *P. nicotianae* in its adaptive evolution to the host.

### Distribution of effectors and their differences in races 0 and 1

Plant pathogens have evolved to secrete effectors, which can manipulate the host immune system and suppress host defense. Based on their target sites in the host plant, effectors can be classified into two classes: (1) apoplastic effectors, which are secreted into plant extracellular spaces; and (2) cytoplasmic effectors, which are translocated into the plant cell. Some effector genes, e.g. *ATR5* in *H. arabidopsidis*, are found to be avirulence genes [46]. These genes are under selective pressure to evade host recognition while maintaining their original functions.

**Fig. 4:**
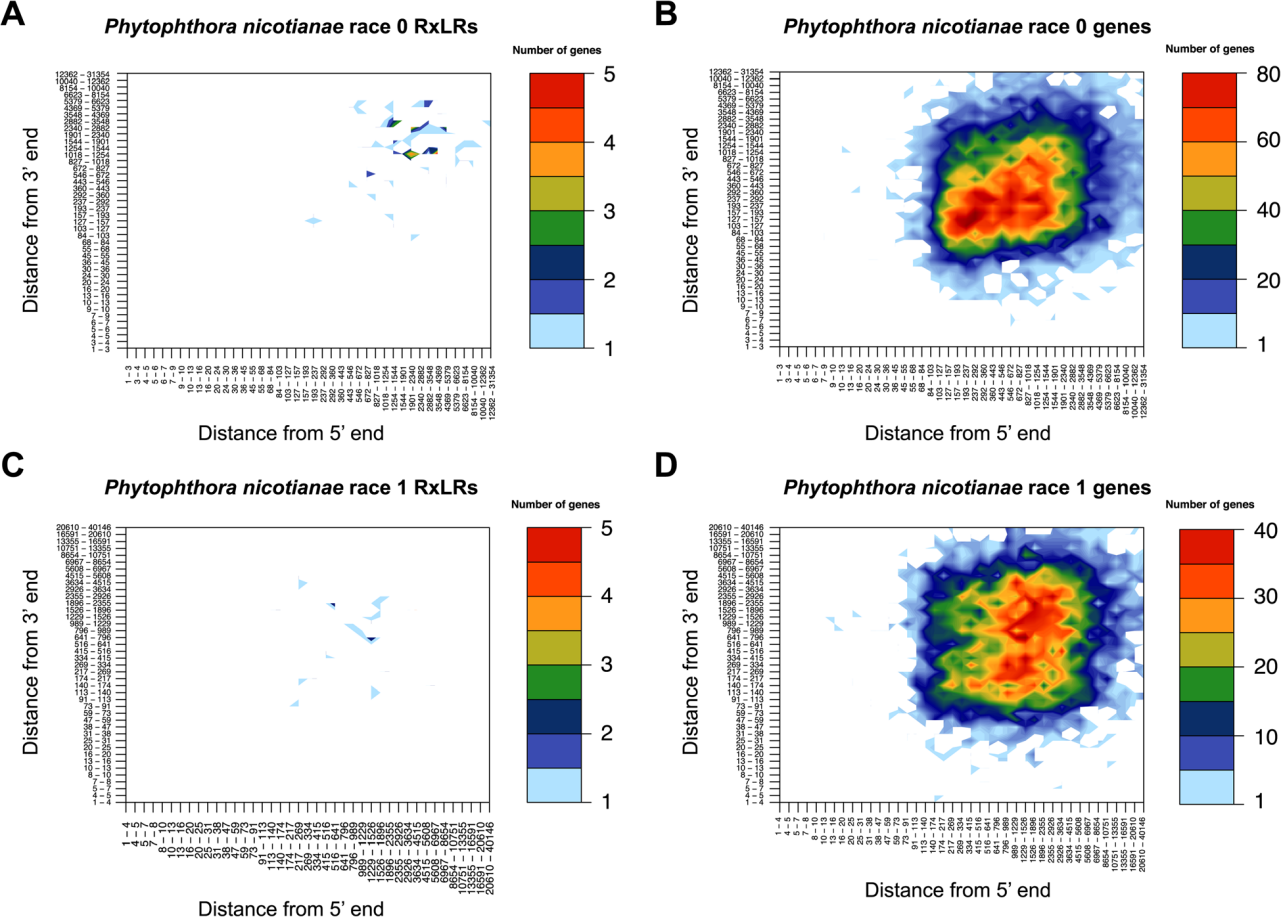
Bin plot showing repeat and gene density distribution. Bins of gene density were sorted and plotted on the basis of 5’ and 3’ intergenic border lengths. The color of each bin represents the number of genes. **a** Distribution in *P. nicotianae* race 0 RxLR effectors. **b** Distribution of *P. nicotianae* race 0 genes. **c** Distribution of *P. nicotianae* race 1 RxLR effectors. **d** Distribution of *P. nicotianae* race 1 genes

RxLR effectors are important cytoplasmic effectors that contain a conserved N-terminal motif (Arg-X-Leu-Arg). The RxLR motif is involved in translocation into host cells [47]. During infection, the RxLR family functions to suppress host immunity. This process usually involves manipulating plant immunity-associated signaling pathways. For example, PexRD2 can perturb MAPKKKε signaling pathways to suppress NB-LRR-mediated immunity in *P. infestans* [48]. In addition, a set of RxLR effectors from *P. infestans* can suppress the signaling pathway induced by flg22, a kind of microbe-associated molecular pattern (MAMP) [49], while some RxLR effectors such as *Avr1b-1* and *Avr1k* can be recognized by NB-LRR immune receptors to confer resistance [50]. Research in 2015 also found that the evolution of RxLR effectors varies between the genus *Phytophthora* and downy mildews: more conserved RxLR effectors were observed in the genus *Phytophthora* [51]. We performed a whole-genome scan for RxLR effector genes in race 0 and race 1. The analysis showed that most RxLR genes were distributed in repeat-rich, gene-sparse regions (Fig. 4), suggesting rapid evolution of RxLR effectors. Specifically, a total of 308 RxLR effector genes were predicted in race 0, and 199 genes in race 1 (Additional files 7 and 8). The difference in RxLR effector gene numbers between race 0 and race 1 may contribute to the variance in the aggressiveness of these pathogens in cultivated tobacco.

Crinkler (CRN) effectors are another important class of effectors that cause leaf crinkling in plants [52]. To investigate CRN effectors in *P. nicotianae* races 0 and 1, we first used EMBOSS getorf (−minsize 300) to extract open reading frames (ORFs) from the whole genome, and then used HMMer (−E 1e-5) with existing profiles [25]. Predicted CRN effectors were filtered by the presence of the LxLFLAK motif. A total of 32 and 26 CRN effectors were annotated in *P. nicotianae* races 0 and 1, respectively. However, the number of CRN effectors may be underestimated, given the model we used [53].

## Availability of supporting data

The genome assembly, annotation and sequencing reads of each sequencing library are available in the NCBI repository, project ID PRJNA294216. The genome assembly and annotation can also be accessed via the *GigaScience* GigaDB database [54].

## Additional files

Additional file 1:
**Statistics of gene lengths of** ***P. nicotianae*** **races 0 and 1, and related species.** Statistics of gene lengths of *P. nicotianae* races 0 and 1, and related species. (XLS 503 kb) Additional file 2:
**Blast hits of ESTs on** ***P. nicotianae*** **races 0 and 1.** Blast results of ESTs on *P. nicotianae* races 0 and 1. (TXT 2187 kb) Additional file 3:
**Positive selected genes between** ***P. nicotianae*** **races 0 and 1.** Positive selected genes between *P. nicotianae* races 0 and 1, identified using KaKs_Calculator. (XLS 518 kb) Additional file 4:
**Venn diagram of gene family clustering between** ***P. infestans*** **,** ***P. ramorum*** **, and** ***P. nicotianae*** **races 0 and 1.** Gene family clustering result from *P. infestans*, *P. ramorum*, and *P. nicotianae* races 0 and 1. A total of 7,552 conserved gene families among the four genomes were identified. (PDF 146 kb) Additional file 5:
**Gene families in** ***P. nicotianae*** **races 0 and 1, and related species.** Total and single copy gene families in *P. nicotianae* races 0 and 1, and related species. (XLS 6019 kb) Additional file 6:
**Pfam annotation result of ABC transporter genes.** Pfam annotation result of ABC transporter genes in *P. infestans*, and *P. nicotianae* races 0 and 1. (TXT 2775 kb) Additional file 7:
**List and amino acid sequences of RxLR and Crinkler effectors.** List and amino acid sequences of RxLR and Crinkler effectors in *P. nicotianae* races 0 and 1. (XLS 228 kb) Additional file 8:
**Comparison of number of effectors between** ***P. nicotianae,*** **and related species.** Comparison of number of RxLR and Crinkler effectors between *P. nicotianae,* and related species. (XLS 59 kb)

## Abbreviations

ABC transporter: ATP-binding cassette transporter
CRN: Crinkler effector
EST: expressed sequence tag
LBA: lima bean agar
LTR: long terminal repeat
ORF: open reading frame
PE: paired-end
RxLR: effectors with Arg-X-Leu-Arg motif
SMRT: single-molecular real-time sequencing
