# Supplementary figures and images for: Genomes and virulence difference between two physiological races of Phytophthora nicotianae
Source: Gigascience. 2016 Jan 28;5:3. doi: 10.1186/s13742-016-0108-7 (PMC4730604; doi:10.1186/s13742-016-0108-7)

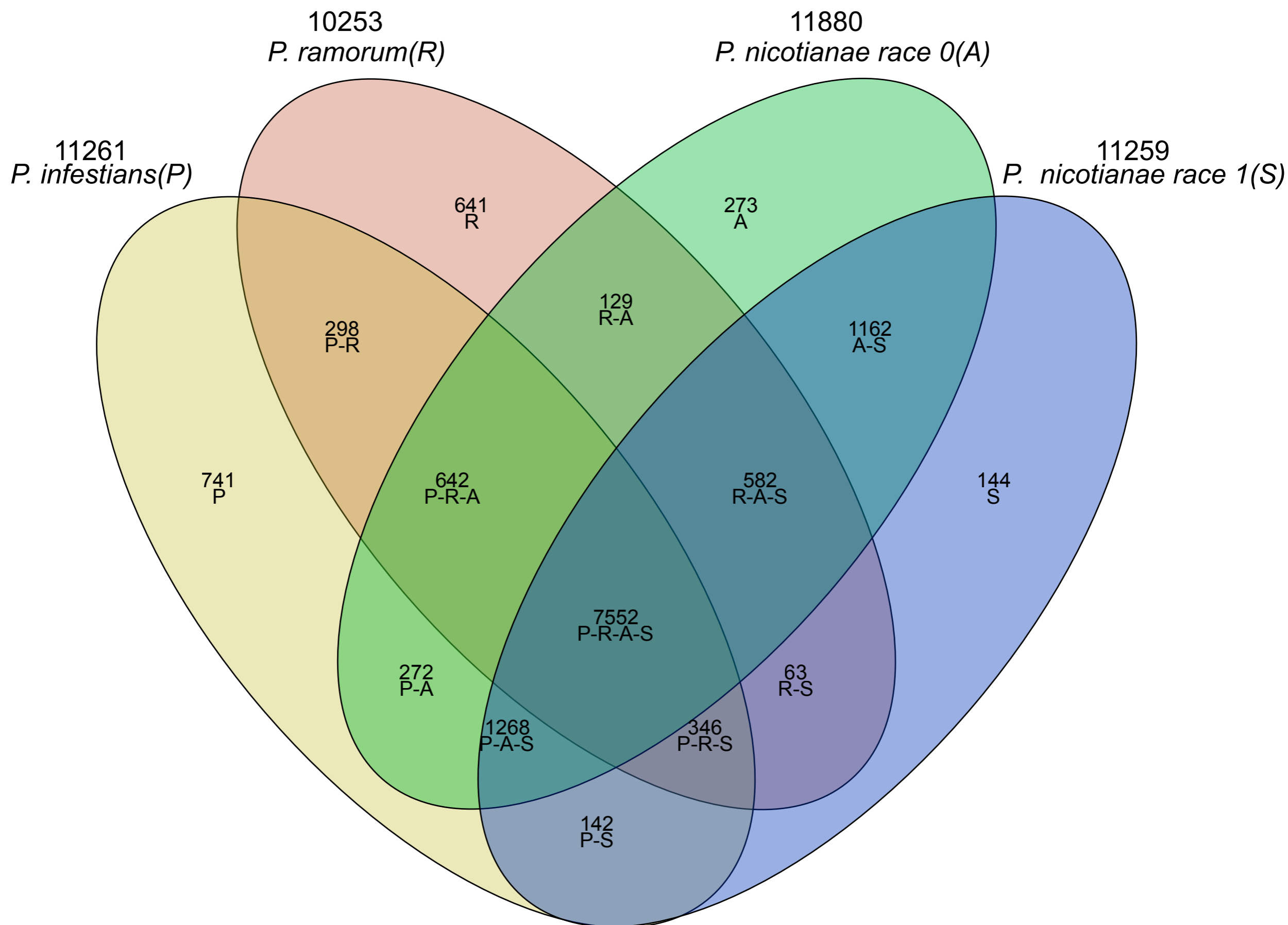

Supplement: Additional file 4: — Venn diagram of gene family clustering between P. infestans , P. ramorum , and P. nicotianae races 0 and 1. Gene family clustering result from P. infestans, P. ramorum, and P. nicotianae races 0 and 1. A total of 7,552 conserved gene families among the four genomes were identified. (PDF 146 kb) [file 13742_2016_108_MOESM4_ESM.pdf]
